# Pervasive HIV recombination limits the utility of circulating recombinant form nomenclature

**DOI:** 10.1371/journal.ppat.1014286

**Published:** 2026-07-13

**Authors:** Heather E. Grant, Abayomi S. Olabode, Dorothea Seiler Vellame, Art F. Y. Poon, Andrew J. Leigh Brown, David L. Robertson

**Affiliations:** 1 Institute of Ecology and Evolution, University of Edinburgh, Edinburgh, United Kingdom; 2 Department of Pathology & Laboratory Medicine, Western University, London, Canada; 3 UK Human Functional Genomics Initiative, University of Exeter, Exeter, United Kingdom; 4 MRC-University of Glasgow Centre for Virus Research, Glasgow, United Kingdom; Boston College, UNITED STATES OF AMERICA

## Abstract

The naming of HIV-1 circulating recombinant forms (CRFs)—descendent viruses from the same intersubtype recombination events, is along with the designation of ‘subtypes’ and ‘groups’, routinely used to track HIV-1 diversity. However, we argue that continuing to designate all detected CRFs as distinct entities is biologically unjustified, as many represent recombinants of limited epidemiological significance. Indeed, the mechanistic underpinning of HIV-1 recombination highlights the arbitrary nature of naming these incidental recombinants, the majority of which are rarely detected again. This underlines the need to prioritise taxonomically meaningful clades, with a focus on biological significance such as emergence events associated with significant epidemiological spread, phenotypic properties or transmission advantage.

## Introduction

When HIV-1 was first sequenced in 1985 [1], it had already undergone over 60 years of evolution [2] before causing a global pandemic. It was not until sequences from central Africa subsequently became available that the enormous scale [3,4] and age [5] of HIV-1 genetic diversity became apparent [6]. Founder effects linked to spread of the main pandemic group (M) from west central Africa [7] have resulted in distinct clades (around 15% divergence; [8]) that are named subtypes A-D and F-H, J and K, with A and F further partitioned into sub-subtypes A1-A6 and F1-F2. In general, the subtypes do not differ clinically in presentation or in response to antiretroviral therapy, but rather reflect their demographic histories. For example, subtype B has been predominantly associated with men who have sex with men and persons who inject drugs in North America and western Europe [9], while subtype C is predominantly found in heterosexual populations in southern Africa [10].

Although direct PCR-sequencing of viral RNA was developed in 1991 [11] lack of automation meant procedures were laborious, and subtype designations were based on sequences of partial regions of the genome or individual genes. For instance, in 1992, the first set of HIV-1 subtypes were designated A–E based on the *env* gene alone—however, it was later noted that subtype E viruses were classified as subtype A based on *gag* [12] and thus most likely not a ‘pure’ subtype. This evidence was substantiated with the emergence of cheaper and more rapid sequencing technologies, where analysis of the whole subtype E genomes established that the *gag* and *pol* regions appeared more like subtype A1 [14,13]. Therefore, this lineage was later designated as the first circulating recombinant form, CRF01_AE, a recombinant of subtypes A and an unknown subtype E [15].

In 1999, an HIV-1 Nomenclature Workshop discussed ambiguities in the HIV nomenclature and formalised the naming of HIV groups, subtypes, sub-subtypes and established circulating recombinant forms (CRF) to describe clades descended from recombination events involving multiple subtypes [15]. To limit identification of incidental recombinants, three or more epidemiologically unlinked genomes with a shared recombination pattern were recommended to designate a CRF. Currently, there are 177 identified HIV-1 CRFs (https://www.hiv.lanl.gov/components/sequence/HIV/crfdb/crfs.comp, accessed 01 June 2026). The genetic diversity within and between CRFs ranges widely: some exhibiting divergence equivalent to subtypes, others being highly related to each other, some CRFs have large numbers of sequences, but many are never sequenced again.

Here, we make the case that while monitoring for clades with novel properties remains important, many CRFs require no special designation. Future designation of HIV-1 clades of interest, whether they are a CRF or not, should focus on evidence for epidemiological significance, as they do for other pathogens.

## Recombination is a continuous process

Mutation and recombination are not entirely independent processes for HIV [16], since both are a consequence of the reverse transcription process [17]. Template switching is an obligate part of reverse transcription [18], and additional template switches can happen multiple times per replication event [19], making recombination more frequent than mutation at this level [20]. While this process serves to repair damaged genomes from the same parental population [21], recombination in individuals with multiple infections can generate unique recombinant forms (URFs). Since the detectability of these events is proportional to the divergence between the parental material, inter-subtype recombination is well documented, while intra-subtype recombination is not.

Signatures of recombination can be detected at both long and short evolutionary time frames. In the more distant past, the SIV from chimpanzee host populations (SIVcpz) from which HIV-1 M descended, is a chimera of two SIVs (SIVrcm and SIVgsn) from different monkey prey species of chimpanzees [22] and has adaptive significance linked to counteracting the antiviral host molecule tetherin [23]. A subsequent deep recombination event within HIV-1 M is supported by differences in the estimated times to the most recent common ancestor across the genome [24].

Global CRF lineages represent a spectrum of older and contemporary recombination events [25], and breakpoints within the same genome can have different ages as sequential recombination events take place [26]. For example, CRF135 is composed of breakpoints between CRF01 and 07, and breakpoints between subtypes C and B in the CRF07 genomic region.

## ‘Pure’ HIV-1 subtypes

All HIV strains are descendants of recombinants to some extent, including the ancestors of subtypes [27], but the ability to detect recombination patterns depends on the divergence between the contributing strains, the time since the event, and the availability of parental reference sequences. This is exemplified by the missing ‘Subtype E’ parent of CRF01_AE [14,13], or by CFR02_AG, which has since been inferred to be the parent of subtype G, not the other way around [28].

Using an unsupervised clustering method that did not rely on pre-defined reference subtypes, Olabode et al. [29] found evidence of extensive recombination in the global diversity of HIV-1 genomes by constructing networks for a series of sliding windows along the alignment, and applying a community detection method (dynamic stochastic block modeling; [30]) to group them according to the relatedness for each window. Recombination was then identified by samples that changed membership between communities. Using this method, the genomic diversity of the M group of HIV-1 was partitioned into 25 clusters, with only 5% of genomes remaining in the same cluster along their entire length. Thus, 95% of genomes across the entire M group were found to be identifiably recombinant.

## Recombination patterns along the genome

**Fig 1:**
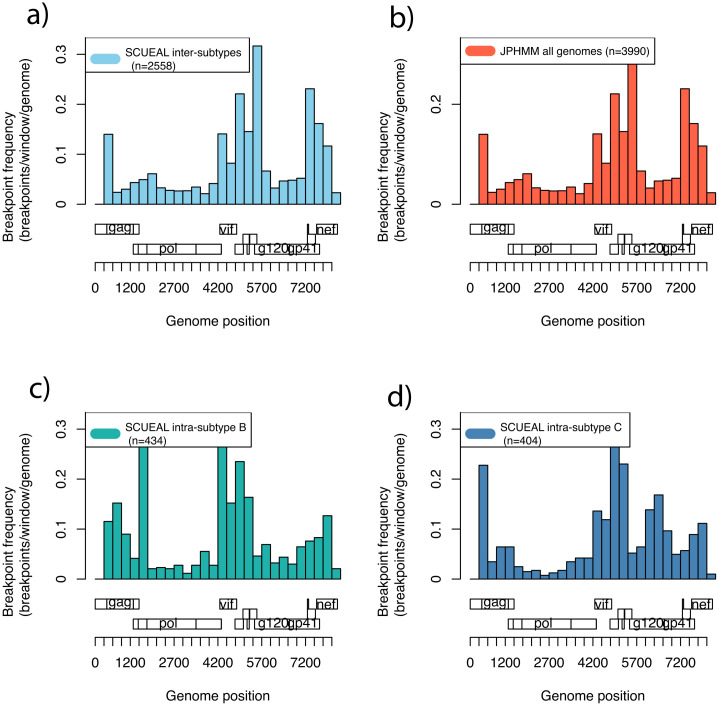
Patterns of recombination along the genome as determined by (a) all available full-length sequences from [29] using SCUEAL (of the *n* = 3900 genomes, *n* = 2588 were recombinant), (b) a similar pattern can be seen using the hidden Markov method-based tool JPHMM. Patterns of recombination within the same subtypes follow similar constraints as seen **(c)** within subtype B, and **(d)** within subtype C.

Recombination can happen anywhere along the genome, determined by several factors, such as sequence similarity [31] and RNA structure [32,33]. Whilst there will be biases in breakpoint frequency along the genome because of these underlying mechanistic processes, selection in the form of viability of a recombinant once generated, will also shape this distribution. A consistent pattern has been observed in multiple studies of breakpoint location, indicating moderate levels of recombination in *gag* and *pol*, very low levels within *env*, and much higher levels at the accessory genes *vif*, *vpr*, *tat*, and *nef* on either side of *env* [29,34–36], which can be observed with phylogenetic and non-phylogenetic based detection tools, between and even within subtypes (Fig 1). The HIV-1 envelope protein is essential for cellular entry with an extremely complex trimer structure, providing a significant functional constraint against disruption by recombination with dissimilar sequence [37–39]. Hence, inter-subtype daughter viruses with recombination breakpoints within *env* may be less successful.

Selection for and against certain breakpoints results in clear hotspots (and coldspots) of recombination that mean similar recombination patterns can arise independently by chance. In Uganda, where 50% of recent genomes are unique recombinants, the envelope gene was found to have recombined intact either as subtype A1 on a subtype D background, or the converse, multiple times [36].

## CRF frequencies

In the Los Alamos HIV Database (accessed 01 June 2026), of all CRF classifications, 82 (46%) had ten or fewer representative sequences, and a large majority (*n* = 146, 82%), had fewer than a hundred. As partial gene sequences are much more common in the database, when restricted to full genomes, 151 (85%) have ten or fewer representatives.

**Fig 2:**
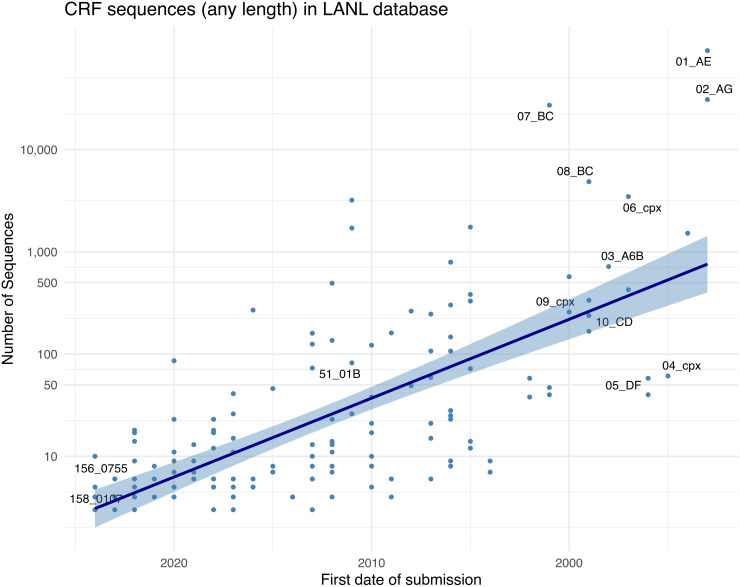
CRF sequence counts in the LANL database (sequences of any length), and the first date they were introduced (accessed 24 November 2024). The number of sequences follows a linear relationship, where the frequency increases 15.6% every year, (linear model, log number of sequences against year, intercept 149, slope = 0.07, $p<2\times 10^{-16}$).

There has been speculation that recombination between subtypes might bring together favourable mutations [40]. But it appears that, in general, the majority of CRFs are not reported after their initial designation. We see a log-linear relationship between the number of representative CRF sequences and the time since their introduction (Fig 2) suggesting that these variants are lost through genetic drift more often than they expand due to any selective advantage.

The three main exceptions are CRF01_AE, CRF02_AG, and CRF07_BC with over 110K, 32K, and 32K sequences, respectively. Together, they alone make up 86% of the CRF sequences available, and each of these CRFs has more numerous representation than the subtypes F, G, H, J, and K combined (28K). This is in part explained by their age; CRF01_AE originated in Africa, before seeding epidemics in Thailand and China, and is a large enough clade that there have been multiple proposals to further stratify it into sub-lineages [41–43]. Similarly, CRF02_AG (composed of subtypes A and G) is a very old and diverse lineage found widely in West and Central West Africa [44,45], while CRF07_BC is a dominant clade in China, presumably linked to its emergence there. Therefore, the origins of these clades are more likely to be similar to those of the subtypes themselves, *i.e.*, reflecting founder effects during the history of the HIV-1 pandemic [46].

## Potential for misleading classification

Different automated subtyping tools include different references, since it quickly becomes cumbersome to include new CRFs as they arise. For example, REGA [47] includes references up to CRF47, and SCUEAL [48] up to CRF51 whereas JPHMM (a hidden Markov Model-based tool) [49] does not include any CRFs in its reference set. In addition, the inclusion of CRF references in the subtyping process can lead to misleading subtyping results as it is difficult to decipher new CRF sections from parts of the genome that resemble the parent.

**Fig 3:**
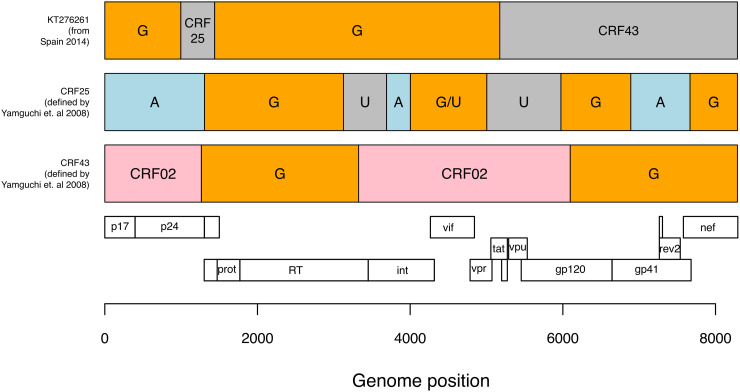
CRFs can make describing HIV-1 diversity problematic. The bottom row in the figure shows an example of a genome from Spain in 2014 [KT276261] with the recombination pattern G/CRF25/G/CRF43 as subtyped by the tool SCUEAL.

Looking at KT276261 as subtyped by SCUEAL (Fig 3), a strict interpretation might suggest this genome is an inter-subtype recombinant with three parents: G, CRF25, and CRF43. However, the section of the genome predicted to be CRF25 has parent subtypes A and G, and the section subtyped CRF43 has parents CRF02_AG and subtype G. Therefore, it could also be the case that KT276261 more resembles a subtype G genome throughout, where parts of the genome are closer to certain reference sections.

Importantly, while subtype classification is straightforward where there is strong geographic structure, in regions where there are multiple subtypes newly circulating, myriad recombinant forms will arise, as is the case in London (UK) [50]. Moreover, where subtypes have co-existed in the same population for several decades, as in Uganda, URFs become the most common form of the virus [36].

## Conclusion

Recombination is a pervasive, ongoing, and even predictable evolutionary process in HIV-1 evolution, such that naming every cluster of circulating recombinants has limited utility. The HIV-1 subtype classification system, whilst imperfect, is widely understood by the community because it reflects founder events that happened before the recorded pandemic [2]. The use of highly curated ‘non-recombinant’ subtype references means that our understanding of HIV diversity is fixed relative to a single point in time.

We do not propose revising the designation of widely established subtypes or CRFs with well-recognised epidemiological relevance. Rather, we suggest that the naming of all new CRFs based on being identified on only three occasions is no longer justified, because many do not have large enough numbers of sequences available to be confident they are of any special functional or epidemiological significance more than incidental transmitted non-recombinant viruses. In addition, tracking and naming every transmitted recombinant overemphasises the readily detectable inter-subtype recombination events, obfuscating the importance of recombination in HIV’s evolutionary history.

Practically, we suggest that use of unsupervised clustering methods, such as the DSBM [29], be used for finding suitable reference sequences. Analyses that require large numbers of sequences to explore the diversity and evolution of HIV (like phylodynamic or clustering studies) would certainly benefit from this approach, as it would include the most appropriate references, whilst also reducing the potentially confounding reticulate evolutionary histories due to inter and intra-subtype recombination. Furthermore, classification of diversity can be updated as more data becomes available, and reflect the frequency of new sequences and the dynamic process of recombination.
